# The transcription factor X-box binding protein-1 in neurodegenerative diseases

**DOI:** 10.1186/1750-1326-9-35

**Published:** 2014-09-12

**Authors:** Julie Dunys, Eric Duplan, Frédéric Checler

**Affiliations:** 1 Institut de Pharmacologie Moléculaire et Cellulaire, UMR7275 CNRS-UNS, Sophia Antipolis, Nice, Valbonne F-06560, France; 2 Université de Nice Sophia Antipolis, Nice, France

**Keywords:** X-box binding protein-1, Unfolded Protein Response, ER stress, Neurodegenerative diseases, Alzheimer’s disease, Parkinson’s disease

## Abstract

Endoplasmic reticulum (ER) is the cellular compartment where secreted and integral membrane proteins are folded and matured. The accumulation of unfolded or misfolded proteins triggers a stress that is physiologically controlled by an adaptative protective response called Unfolded Protein Response (UPR). UPR is primordial to induce a quality control response and to restore ER homeostasis. When this adaptative response is defective, protein aggregates overwhelm cells and affect, among other mechanisms, synaptic function, signaling transduction and cell survival. Such dysfunction likely contributes to several neurodegenerative diseases that are indeed characterized by exacerbated protein aggregation, protein folding impairment, increased ER stress and UPR activation. This review briefly documents various aspects of the biology of the transcription factor XBP-1 (X-box Binding Protein-1) and summarizes recent findings concerning its putative contribution to the altered UPR response observed in various neurodegenerative disorders including Parkinson’s and Alzheimer’s diseases.

## Introduction

Endoplasmic Reticulum (ER) is the compartment where transmembrane and secreted proteins transit to be matured and properly folded before routing to their final location. Function and homeostasis of this structure are crucial for cell fate. When ER is subjected to a stress, a protein overload or any dysfunction, an adaptative response, called Unfolded Protein Response (UPR) is initiated in order to restore ER homeostasis (for review see [1,2]). UPR failure results in the activation of an apoptosis-dependent cell death. The UPR activates the transcription of several genes that are involved in the reduction of protein synthesis as well as in the chaperoning and degradation of misfolded or unfolded proteins. Such process involves interplay between distinct signaling pathways mediated by several transmembrane sensors, namely PKR-like ER kinase (PERK), Activating Transcription Factor 6 (ATF-6) and Inositol Requiring Enzyme 1 alpha (IRE1α). The most conserved of these pathways implies the activation of IRE1α, a Ser/Thr protein kinase that also harbors an endoribonuclease activity. Once autoactivated, IRE1α induces an unconventional splicing of the mRNA encoding the X-box Binding Protein-1 (XBP-1) transcription factor, which subsequently regulates the transcription of genes involved in ER homeostasis. Recent studies have delineated novel XBP-1 target genes and have documented additional ER stress- and UPR- independent functions. Here we first briefly describe some aspects of XBP-1 biology and report on the experimental clues of its implication in various metabolic and inflammatory disorders as well as in several pathologies including cancer and neurodegenerative diseases such as amyotrophic lateral sclerosis, Huntington’s, Parkinson’s and Alzheimer’s diseases.

### X-box binding-protein 1 discovery

XBP-1 has been first described more than two decades ago by the group of Dr Laurie Glimcher who worked on MHC class II genes regulation [3]. Her group discovered and characterized XBP-1 as a new member of the basic region leucine zipper protein family (bZIP). This family of transcription factors is involved in a wide spectrum of physiological and pathological functions. Interestingly in yeast, hac-1 that belongs to the bZIP family, contributes to UPR activation in response to environmental stress [4,5]. In eucaryotic cells, endoplasmic reticulum stress is coupled to the splicing and thereby, activation of XBP-1 that appears as the mammalian counterpart of hac-1 [6].

### XBP-1 is activated by an unconventional splicing mechanism

**Figure 1:**
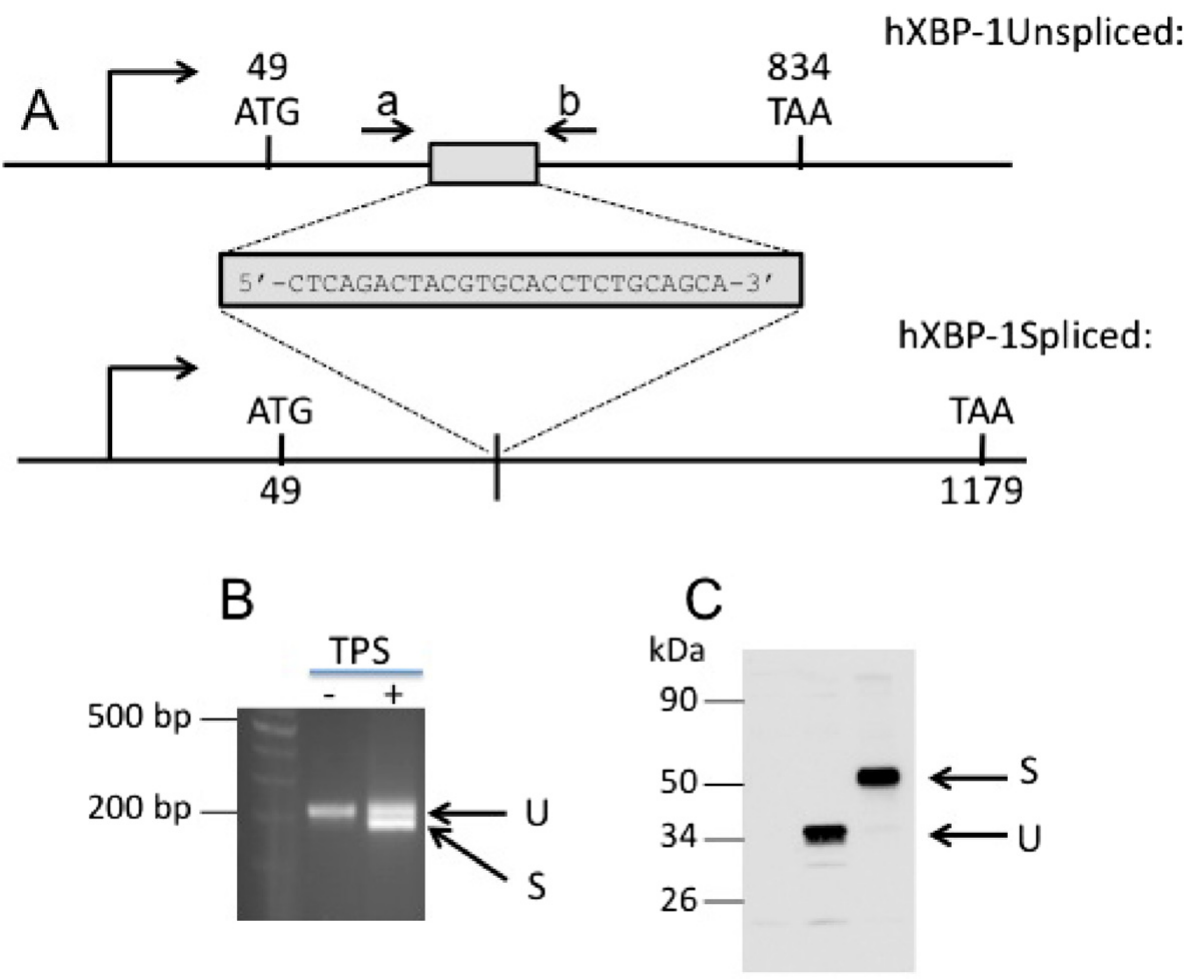
**Unconventional splicing of XBP-1.***Xbp-1* gene is constitutively transcribed into a mature mRNA (hXBP-1Unspliced; panel **A)** The resulting mRNA (U, panel **B**) contains a 26 nucleotides long intron that is translated into an unstable and short 261 amino acids-long protein (U, panel **C**). Upon ER stress, IRE1 autoactivates and triggers an unconventional excision of the 26 nucleotides long intron. This splicing results in a lower molecular weight of the semi quantitative PCR fragment (S, panel **B**) yielded by primers a and b used for PCR (flanking the splicing region of XBP-1, see panel **A)** but results in a modified reading frame resulting in a longer protein (S, panel **C**).

XBP-1u (unspliced) mRNA is produced constitutively and yields a protein that is rapidly degraded in physiological conditions by the proteasome machinery [7]. When ER stress occurs, the IRE1α kinase is activated through autophosphorylation and acts as a stress sensor and transducer. IRE1α endoribonuclease activity then removes a 26 nucleotides intron from XBP-1u mRNA coding sequence inducing a frame shift [8,9]. Thereafter, subsequent processed mRNA is translated onto a more stable 376 amino acids-long isoform XBP-1s (spliced), which bears the transcriptional activity (Figure 1).

This unconventional splicing mechanism is commonly triggered by misfolded proteins overload but not only. Dysregulations in lipid biosynthesis also induce an ER stress response. IRE1α is able to appreciate imbalance in membrane lipid composition and membrane fluidity modulation. In such conditions, after homodimerization and autophosphorylation, IRE1α activates the unconventional XBP-1 mRNA splicing inducing transcription of genes implicated in lipid biogenesis. Moreover, it is interesting to note that UPR regulates transcription of genes involved in the lipid biosynthetic pathways [10].

Beside its effect on XBP-1 splicing-dependent activation, IRE-1α can process and/or degrade several other mRNAs and miRNAs through a mechanism called RIDD (Regulated IRE1α Dependent Decay) [11]. IRE1α also contributes in additional cellular pathways through mechanisms that do not involve its endoribonuclease activity but its kinase activity.

### XBP-1 function in multiple signaling pathways and diseases

Mammalian XBP-1 protein is widely expressed in adult tissues and has a major role in development. The question of XBP-1 function was addressed by examining the contribution of its endogenous counterpart in mice knock-out model [12]. Unfortunately, the homozygous knock-out is lethal by day 10.5 to 14.5 *in utero* while heterozygotes did not develop any defect. This premature lethality is explained by heart dysfunction due to an acute cellular necrosis of cardiac myocytes.

Another developmental function of XBP-1 concerns transition of mature B cells to antibody-secreting plasma cells. XBP-1 transcripts are upregulated by stimuli inducing plasma cell differentiation [13]. XBP-1 expression is driven by IgM production and enhances immunoglobulin secretion [14]. More largely, XBP-1 is involved in the development of secretory tissues. Its transcriptional activity is essential for hepatocyte growth. Thus, aside their growth retardation, XBP-1^-/-^ embryos develop hypoplastic livers [15]. At the adulthood, XBP-1 conditional knock-out revealed that the transcription factor regulates genes implicated in hepatic lipogenesis [16].

ER stress and defects in the UPR adaptative response have been linked to a high number of pathologies such as metabolic disorders, immune and inflammatory diseases, cardiovascular pathologies, cancers, and brain disorders (Table 1). Moreover, XBP-1 has a growing number of targets and functions related more or less to its role in ER-stress regulation. Transcriptional targets of XBP-1s already include genes implicated in support of ER function i.e. ER chaperones such as the Protein Disulfide Isomerase (PDI) [17], GRP78 BiP co-chaperone, Erdj4 [18], ERAD components such as the E3 ubiquitin ligase HRD1 [19] or C/EBP alpha which is an inducer of adipogenesis [20]. Other more unexpected targets connect spliced XBP-1 to DNA damage and repair pathways, myodegenerative and neurodegenerative diseases [21]. XBP-1s binding site contains a consensus sequence consisting of an ACGT motif [18].

**Table 1:** Evidences of XBP-1 implication in multiple pathologies

**Pathologies**	**Evidence**	**References**
*Metabolic disorders*		
*- Diabetes*	XBP-1 influences glucose homeostasis	[22-24]
*- Non-alcoholic fatty liver disease*	XBP-1/PDI/MTP axis activation	[25]
	TLR4 dependent XBP-1 activation by HFHC diet	[26]
*Inflammatory diseases*		
*-* Rheumatoid arthritis	XBP-1 upregulation	[27]
*-* Inflammatory bowel disease	*XBP-1* gene single-nucleotide polymorphisms	[28]
*Cancers*		
*-* Lymphoid malignancies	XBP-1 inhibition reduces leukemic progression	[29]
*-* Breast cancers	Activation of UPR and upregulation of XBP-1	[30-32]
*-* Colorectal cancers	XBP-1 overexpression in adenomas and adenocarcinomas	[33]
*Neurodegenerative diseases*		
*-* Huntington’s disease	XBP-1 invalidation protects over HD	[34]
*-* Alzheimer’s disease	XBP-1 overexpression in AD patients	[35-37]
	*XBP-1* polymorphism associated to AD risk	[38]
*-* Parkinson’s disease	Implication of IRE1-XBP-1 in dopaminergic neurons survival	[39-41]
*-* Amyotrophic lateral sclerosis	Increase in XBP-1 splicing in ALS models	[42,43]

### XBP-1 in metabolic disorders

Several lines of evidence suggest that obesity and type-2 diabetes share cellular conditions that trigger ER stress. Pharmacological stimulation of ER stress in liver cells inhibits insulin action [22]. Mice developing a diet-induced obesity present an increase in ER stress dependent XBP-1 splicing. Moreover, XBP-1 signaling is involved in insulin sensitivity since XBP-1^+/-^ mice present glucose homeostasis impairment and are more susceptible to develop a diet-induced insulin resistance.

At the molecular level, two different studies recently brought explanations linking ER stress and PI3K pathway through XBP-1 nuclear translocation mechanism [44,45]. Insulin signaling disrupts the complex between two subunits of the Phosphatidylinositol-4,5-bisphosphate 3-kinase (PI3K), p85α and p85β, and induces the formation of an heterodimer complex between these subunits and XBP-1s. Formation of this heterodimer allows XBP-1 nuclear translocation.

Interestingly, XBP-1 also regulates glucose homeostasis through transcription independent mechanism [23]. An XBP-1s mutant defective for DNA binding is able to increase glucose tolerance. Spliced and activated XBP-1 isoform (XBP-1s) induces proteasomal degradation of Forkhead box O1 (FoxO1), thus highlighting for the first time a function of XBP-1 independent of its transcriptional activity [23,46].

### XBP-1 in inflammatory diseases

Discovery of several single-nucleotide polymorphisms on *XBP-1* gene related to inflammatory bowel disease (IBD) has pointed out a link between ER stress and tissue specific inflammatory pathologies [28]. Intestinal-specific XBP-1 invalidation results in a defective antimicrobial response as well as ER stress induction and inflammatory response.

It has been postulated that XBP-1 could be activated independently of ER stress, UPR mechanism and IRE1α activation, through the Toll-Like Receptor (TLR) pathway. This pathway is primordial for cytokine secretion and has been described in synovial fibroblasts of patients suffering from active rheumatoid arthritis, a joint inflammatory disease [27]. While XBP-1 was upregulated in synovial fibroblasts of Rheumatoid Arthritis (RA) patients, other UPR markers were largely down-regulated, suggesting the uncoupling between the two signaling pathways. Two TLR isoforms, TLR4 and TLR2, induce production of pro-inflammatory cytokines such as tumor necrosis factor alpha (TNFα) and interleukine-6 (IL-6). In turn, TNFα potentiates XBP-1 splicing therefore holding a positive feedback loop [27].

### XBP-1 in cancer

XBP-1 is expressed ubiquitously and is increased in many types of cancers as is the case for several downstream targets of UPR. The pathways through which IRE1α/XBP-1 branch is involved in progression of different cancers is still unknown and few hypotheses begin to emerge. XBP-1 transcription factor is implicated in a number of pathways related to tumorigenicity, such as apoptosis and mitochondrial permeability, drug resistance and drug induced cell cycle arrest (for review see [47]). Moreover, decrease in XBP-1 expression appears to potentiate cell sensitivity to hypoxia. The IRE1α-XBP-1 branch of the UPR has been implicated in regulation of proliferation through the modulation of cyclin A1 [48].

The role of XBP-1 in progression of mammary epithelial cell cancer has recently been investigated. XBP-1 transcriptional activity drives Triple-Negative Breast Cancer (TNBC) tumorigenicity and activation of the transcription factor is correlated with a poor prognosis [30]. XBP-1 silencing triggers an inhibition of cell growth and tumor invasiveness. The mechanism of XBP-1 implication in TNBC progression involves interaction with another transcription factor, HIF-1α [30].

One of the developmental functions of XBP-1 concerns B cell differentiation [13]. Paradoxically, XBP-1 appears to intervene also in chronic lymphocytic leukemia, since XBP-1 inhibition interferes with leukemia cells and lymphoma survival [29].

Overall, many studies bring together evidences pointing out that XBP-1 is of particular interest regarding its potential as an anti-cancer therapeutic target.

### XBP-1 in neurodegenerative diseases

The accumulation and aggregation of misfolded proteins is a common feature for neurodegenerative disorders. Those aggregates are harmful for neurons and lead to activation of UPR signaling pathways in order to restore ER homeostasis. However, when the UPR process fails in this task, a prolonged ER stress could trigger neuronal cell death as it is the case in several neurodegenerative diseases. The IRE1α/XBP-1 branch of UPR has been implicated in several neurodegenerative disorders, some of which are developed hereafter.

### XBP-1 in Amyotrophic Lateral Sclerosis

Amyotrophic Lateral Sclerosis (ALS) is a dramatic and lethal adult-onset degenerative disease characterized by muscular weakness, atrophy and paralysis due to brain stem, spinal cord, cortico-spinal tract, primary motor cortex motoneurons neurodegeneration and degradation of the neuromuscular junctions [49]. Several data incriminate ER stress and aggregation of misfolded proteins in ALS etiology.

Rodents expressing mutations in the *SOD1* gene, which is involved in genetic familial cases of ALS, show an activation of the three branches of the UPR and an increase in the IRE1α dependent unconventional splicing of XBP-1 mRNA [42]. Unexpectedly, selective deficiency of XBP-1 in the nervous system of a rodent model of familial ALS triggers a decrease in SOD1 accumulation through an autophagy-dependent mechanism, which induces motoneurons survival [43].

### XBP-1 in Huntington’s disease

Huntington’s disease (HD) is an inherited genetic disorder that progressively causes nerve cell degeneration. The dominant genetic alteration is a CAG trinucleotide repeat in the *IT15* gene leading to the expression of an expanded polyglutamine (polyQ) track at the N-terminus of the Huntingtin protein (Htt) [50,51]. The length of this polyQ motif, which usually does not exceed 40 glutamines in healthy persons, is correlated to the severity of the pathology [52]. Moreover, the number of polyglutamine repeats drives the propensity of Htt to aggregate. Folding abnormalities are common in trinucleotide repeat disorders and often linked to neuronal cell death. Polyglutamines expansions on mutant Htt protein impair several physiological functions of the protein. The question of the role of the Htt aggregates in the pathology – are they a cause or a consequence – remains to be determined. However, ER stress and UPR activation have been reported in HD models and markers of these two pathways have been found after *post-mortem* analysis of HD patient’s brains [34,53]. Moreover, polyglutaminated mutant Htt appears to inhibit ERAD process therefore enhancing ER stress induced apoptosis [54].

Literature offers contrasting results about the implication of XBP-1 in aggregation of mutant Htt. On the one hand, an increase in XBP-1 mRNA unconventional splicing has been noticed in transgenic animals that express a mutant Htt. In addition, conditional XBP-1 deficient mice appear to be less prone to develop the pathology and present less neuronal cell death and reduced motor deficits [34]. Interestingly in this study, XBP-1 implication in Huntington’s disease seems to be independent of its function in ER stress regulation. XBP-1 depletion tends here to influence mutant Htt degradation through an autophagy dependent mechanism [34]. On another hand, a study performed in adult mice through adeno-associated viral (AAV) specific delivery into *striatum* of an active form of XBP-1 showed a reduction of Htt aggregation and inclusions formation [55].

### XBP-1 in Alzheimer’s disease

Alzheimer’s disease (AD) is the most common form of age-related dementia worldwide. The pathology is characterized by two morphological stigmata: senile plaques consisting of extracellular aggregates mainly composed of a set of amyloid-beta-related peptides (Aβ) and neurofibrillary tangles that are intracellular aggregates of a hyperphosphorylated protein, the microtubule associated protein Tau. These two histopathological markers are accompanied by oxidative stress, neuroinflammation, synaptic deficits and neuronal cell death.

Several studies have suggested an induction of ER stress and an activation of UPR signaling pathways in Alzheimer’s disease [56]. Increases in markers such as GRP78 and phospho-PERK have been described in cortex and hippocampus of AD patients [57,58]. In familial genetic cases of AD, ER stress induction and UPR attenuation have been described [59] while in sporadic cases of the pathology, ER stress is due to a reduction in Protein Disulfide Isomerase (PDI) activity [60]. However, such ER stress activation has not been recovered in aged Tg2576 mice, a transgenic mouse model that develops plaques and synaptic failures but lack the Tau dependent counterpart of the pathology [37]. XBP-1 mRNA unconventional splicing appears to be increased in the cortex of AD patients as well as PDI expression [37]. Moreover, it has been suggested that the polymorphism -116C/G of *XBP-1* gene could increase the susceptibility to develop Alzheimer’s disease in a Chinese population [38].

XBP-1 spliced isoform has been shown to mediate protective effects against amyloid-β peptide and amyloid oligomers induced toxicity [35]. Therefore, XBP-1s tends to impair cytoplasmic calcium accumulation through the regulation of ryanodine calcium channel RyR3. Interestingly, XBP-1 unconventional splicing was potentiated by amyloid-β peptide and Aβ oligomers in mammalian neurons cultures as well as in drosophila neurons suggesting a highly conserved mechanism [35].

Recently, a screening approach carried out in order to identify transcription factors implicated in regulation of two secretases involved in the amyloid precursor protein (APP) processing pathways, ADAM10 (A Disintegrin And Metalloproteinase 10) and BACE1 (βAPP Cleaving Enzyme 1) [61] pointed out the entailment of XBP-1 in ADAM10 regulation. Thus, this study revealed that XBP-1s is induced at an early time point in two transgenic mouse models mimicking in part the pathology [36]. ADAM10 expression is transcriptionally modulated by XBP-1 in neuronal cells and such modulation can be achieved by pharmacological induction of ER stress [36].

One of the transcriptional targets of XBP-1 during ER stress is the protein HRD1 which is involved in ERAD process. HRD1 expression has been found consequently diminished in AD brains [62]. Interestingly, HRD1 binds to APP and promotes its ubiquitination and subsequent proteasomal degradation. Therefore in addition to its role on ADAM10 expression and through activation of HRD1 and degradation of APP, XBP-1 indirectly modulates amyloid-β production.

### XBP-1 in Parkinson’s disease

Parkinson’s disease (PD) is a neurodegenerative disorder characterized by specific death of dopaminergic neurons of the substantia nigra *pars compacta* (SNpc). At the histological level, affected neurons present intraneuronal inclusions, called Lewy bodies that are composed predominantly of aggregated α-synuclein protein.

Involvement of ER stress and UPR activation in Parkinson’s disease has been described in pharmacological models of the pathology [63] as well as in patient’s brain [64]. Moreover, characteristic defaults found in PD models such as mitochondrial dysfunction, protein misfolding, protein aggregation and degradation impairment are known to trigger ER stress. However, contribution of ER stress to the disease is not very well understood but several studies have examined this possibility. In human brains, UPR activation has been investigated in the substantia nigra. It appeared that neurons containing high concentration of α-synuclein were also positive for phospho-PERK and PD patient’s brain present an activation of the PERK-eIF2α pathway of the UPR [64].

The implication of a second branch of the UPR in dopaminergic neurons survival has been examined. The IRE1α-XBP-1s dependent pathway was activated in a model of specific dopaminergic neurodegeneration induced by MPTP (1-methyl-4-phenyl-1.2.3.6-tetrahydropyridine) [65]. Adenoviral mediated expression of XBP-1s in MPTP-treated mice tend to foil dopaminergic neurons death, suggesting that XBP-1s has protective effects against PD mimetic insults. Another study confirmed XBP-1 protective effects and published recently similar observations. Local stereotaxic delivery of XBP-1s in the substantia nigra induces a neuroprotection against 6-hydroxydopamine (6-OHDA) exposure [39]. Moreover, selective invalidation of XBP-1 in dopaminergic neurons of the substantia nigra *pars compacta* triggers chronic ER stress and neurodegeneration of the neurons targeted [39]. It has also been postulated that α-synuclein aggregates promote XBP-1 splicing and activation in human neuroblastoma [41].

We have recently uncovered a novel transcriptional target of XBP-1s, the protein DJ-1 [40]. Mutations on DJ-1 gene (*Park7*) have been associated with autosomal recessive early-onset forms of the disease [66]. We first demonstrated that DJ-1 expression is under control of parkin (PK) another protein involved in familial recessive cases of PD. Parkin, which has been largely studied for its role as an E3-ubiquitin ligase [67] possesses another function as a transcription factor [68,69]. Thus, we established that parkin represses the transactivation of the p53 promoter [68]. Interestingly we showed that p53 acts as an upstream negative regulator of XBP-1 [40]. Furthermore, we identified a XBP-1 consensus binding motif within the DJ-1 promoter sequence that is conserved in several species. Overall, our data suggested that parkin could control DJ-1 expression through a cascade involving two intermediates transcription factors p53 and XBP-1 [70]. Moreover, we have shown that this indirect control of DJ-1 by parkin can be abrogated by autosomal recessive parkin mutations implicated in familial cases of PD [40].

Altogether, these different studies suggest that targeting the UPR and for example modulating XBP-1s expression through gene transfer may have therapeutic potential benefits in order to treat Parkinson’s disease.

## Conclusion

Regulation of ER homeostasis is a key feature in several pathological conditions. When UPR fails to buffer ER stress imbalance, it turns out to induce cell death. This point is critical in neurodegenerative diseases since neuronal cell death is highly detrimental. XBP-1 has important implications in ER stress induced transcriptional regulations.

It has been pointed out that XBP-1 could also have functions in modulation of metabolism, inflammation or lipogenesis in a transcription factor independent manner. In some neurodegenerative disorders, XBP-1 implication is also independent of ER stress and UPR activation.

Overall, XBP-1 appears as a pleiotropic transcription factor regulating a wide set of proteins involved in various functions linked to or independent of UPR and ER stress and therefore, could be seen as a putative target of therapeutic strategies aimed at interfering with distinct pathologies.

## Abbreviations

6-OHDA: 6-hydroxydopamine; Aβ: Amyloid-β peptide; AD: Alzheimer’s Disease; ADAM10: A Disintegrin And Metalloproteinase 10; ALS: Amyotrophic Lateral Sclerosis; APP: Amyloid Precursor Protein; ATF6: Activating transcription factor 6; BACE1: βAPP Cleaving Enzyme 1; BiP: Binding immunoglobulin protein; ERAD: Endoplasmic Reticulum Associated protein Degradation; FoxO1: Forkhead box O1; HD: Huntington’s Disease; HFHC: High Fat High Cholesterol; HIF1: Hypoxia inducible factor 1; Htt: Huntingtin; IRE1: Inositol-requiring endonuclease 1; MPTP: 1-methyl-4-phenyl-1.2.3.6-tetrahydropyridine; PD: Parkinson’s Disease; PDI: Protein Disulfide Isomerase; PERK: PKR-like ER kinase; PI3K: Phosphatidylinositol-4,5-bisphosphate 3-kinase; RIDD: Regulated IRE1α Dependant Decay; RyR3: Ryanodine receptor 3; SOD1: Sodium dismutase 1; TLR4: Toll-Like Receptor 4; TNBC: Triple-Negative Breast Cancer; TNFα: Tumor Necrosis Factor alpha; UPR: Unfolded Protein Response; XBP-1: X-box Binding Protein-1.

## Competing interests

The authors declare that they have no competing interests.

## Authors’ contributions

Final manuscript version has been approved by all the authors.
